# Sequencing of Bacterial Genomes: Principles and Insights into Pathogenesis and Development of Antibiotics

**DOI:** 10.3390/genes4040556

**Published:** 2013-10-14

**Authors:** Eric S. Donkor

**Affiliations:** Department of Microbiology, University of Ghana Medical School, P. O. Box 4236, Accra, Ghana; E-Mail: ericsdon@hotmail.com; Tel.: +233-302-665404

**Keywords:** genome, pathogenesis, bacteria, sequencing, antibiotics

## Abstract

The impact of bacterial diseases on public health has become enormous, and is partly due to the increasing trend of antibiotic resistance displayed by bacterial pathogens. Sequencing of bacterial genomes has significantly improved our understanding about the biology of many bacterial pathogens as well as identification of novel antibiotic targets. Since the advent of genome sequencing two decades ago, about 1,800 bacterial genomes have been fully sequenced and these include important aetiological agents such as *Streptococcus pneumoniae*, *Mycobacterium tuberculosis*, *Escherichia coli* O157:H7, *Vibrio cholerae*, *Clostridium difficile* and *Staphylococcus aureus*. Very recently, there has been an explosion of bacterial genome data and is due to the development of next generation sequencing technologies, which are evolving so rapidly. Indeed, the field of microbial genomics is advancing at a very fast rate and it is difficult for researchers to be abreast with the new developments. This highlights the need for regular updates in microbial genomics through comprehensive reviews. This review paper seeks to provide an update on bacterial genome sequencing generally, and to analyze insights gained from sequencing in two areas, including bacterial pathogenesis and the development of antibiotics.

## 1. Introduction

Bacterial diseases constitute an important cause of morbidity and mortality among humans and also animals. Pathogenic bacteria include a wide range of organisms which employ varied mechanisms in pathogenesis [1]. Design of therapeutic interventions against bacterial diseases requires a good understanding of the mechanisms by which these pathogens employ in causing diseases [2]. Unfortunately, the pathogenesis of many pathogens is poorly understood. The advent of genome sequencing coupled with advances in bioinformatic analysis to model genome data, promises invaluable insights into bacterial pathogens, including, their evolution, ecology, pathogenesis, and the design of related therapeutic interventions. So far, about 1,800 bacterial genomes have been fully sequenced and these cover most of the major bacterial pathogens [3,4,5]. 

This review paper analyzes insights gained from the applications of genome sequencing in two areas of biomedical science, including the mechanisms by which bacteria cause disease and the development of antibiotics. 

## 2. Brief Overview of Bacterial Pathogenesis

Pathogenic bacteria possess certain features referred to as virulence determinants which enable them to cause disease in susceptible hosts [1,6]. These features include

- Adherence factors: these are attachment devices such as pili, fimbriae, and adhesins which enable pathogenic bacteria to adhere to host cells. For example *Escherichia coli*, a common aetiological agent of urinary tract infection, attaches to uroepithelial cells by means of pyelonephritis-associated pili [1,6,7]. In the pathogenesis of gonorrhea, *Neisseria gonorrhoeae* attaches to mucosa epithelial cells by means of type IV pili and an outer membrane adhesion, *Opa* [1,6,8].
- Toxin production: Various exotoxins are elaborated by pathogenic bacteria, which include cytotoxin, enterotoxin and neurotoxin [9]. *Corynebacterium* *diptheriae*, the aetiological agent of diphtheria produces a heat labile cytotoxin. In the presence of NAD, Fragment A component of the toxin inactivates EF-2, causing the inhibition of polypeptide elongation and therefore protein synthesis [10]. *Vibrio cholerae*, the cause of cholera produces an enterotoxin which activates the adenylate cyclase enzyme in intestinal mucosa cells resulting in high levels of intracellular cAMP, and also the secretion of water and ions into the small intestine lumen [1,11,12]. Tetanus is mediated by a neurotoxin produced by *Clostridium tetani*; the toxin prevents the release of γ-aminobutyric acid thereby causing spastic paralysis [13]. In addition to exotoxins, endotoxin may be produced by Gram-negative bacteria, especially when they lyse. Endotoxins are essentially lipopolysaccharides which can induce overwhelming inflammatory responses and are important in sepsis and septic shock [7,9].
- Invasins: these include a wide range of extracellular enzymes or proteins which enable bacterial pathogens to invade host tissues. Using *Staphylococcus aureus* as an example, this organism produces a wide range of invasins including hyaluronidase which breaks down hyalauronic acid of connective tissues, DNases which break down DNA, haemolysins which split red blood cells, staphylokinase which activates plasminogen to plasmin, an enzyme digesting fibrin clots [1,14,15]. Several other invasins such as proteases, lipases, nucleases, collagenase and elastase are produced by *Staphylococcus aureus* [1,14].
- Capsule: bacterial capsule contributes to the virulence of some bacteria such as *Streptococcus pneumoniae* and *Neisseria meningitidis* by helping them resist phagocytosis of the host defense system [1].

Some bacterial pathogens are able to evade host defenses, including phagocytosis, complement, and immune response through various mechanisms [16,17,18]. For example, *Streptococcus pyogenes* produces hyaluronic acid capsule which covers and shields most of the antigenic proteins of the organism from the host immune system [16]. Since the human connective tissue also contains hyaluronic acid, this limits antibody response against the *Streptococcus pyogenes* capsule. *Haemophilus influenzae* is able to modify its lipopolysaccharide (principal target of complement) by the attachment of sialic acid to its O antigens resulting in resistance to membrane attack complex [17]. *Yersinia* species, which include important disease agents such as the causative agent of plague (*Y. pestis*), employ their type III secretion system to transfer T3SS effectors that neutralize phagocytic activity [18].

Bacterial pathogenesis is an interplay of bacterial virulence determinants and the host immune response, and the actual mechanism involved varies from one organism to another [1]. A bacterial pathogen may not need all the virulence features mentioned above to cause disease in its host. For instance in botulism, a kind of food poisoning, the clinical condition is caused solely by a neurotoxin produced by *Clostridium botulinum*, the etiological agent of the disease [19]. 

Various genetic mechanisms are known to play important roles in bacterial pathogenesis. Generally, horizontal gene transfer plays an important role in the acquisition of virulence determinants [20]. Some bacterial pathogens, such as *Shigella* spp. may show increased pathogencity as a result of gene loss or modification of some genes [21]. In some cases, as with *Neisseria gonorrhoeae*, programmed genomic alterations involving site-specific recombination systems are induced resulting in an antigenic phase variation in cell surfaced expressed genes [22]. In some bacterial pathogens such *Pseudomonas* *aeruginosa*, mutation rate may be increased by error prone repair of DNA mismatches as a means of adaptation to a new environment or host [22]. Through whole genome sequencing of bacterial pathogens deeper insights have been gained into some of these genetic mechanisms (Section 5.4).

## 3. Brief Overview of the Interactions between Bacteria and Antibiotics

Antibacterial agents or antibiotics constitute the main form of treating infections caused by bacterial pathogens, and they affect the pathogens by either selectively inhibiting their growth (static effect) or killing them (cidal effect) [23]. Since the discovery of penicillin (the first antibiotic) in 1928, a wide range of antibacterial agents have been discovered [24]. Overall, the mechanisms of action of antibacterial agents involve targeting five sites in the bacterial cell which include:

- Inhibition of cell wall synthesis: The most important drugs in this group are the β-lactams that bind and inhibit penicillin binding proteins which catalyze formation of peptidoglycan cross-links in the bacterial cell wall [23,25,26,27]. This action weakens the cell wall of the bacterium causing cytolysis [27].
- Inhibition of protein synthesis: drugs of this class include aminoglycosides, tetracyclines, macrolides, and chloramphenicol; they act at the level of the ribosome and interfere with protein synthesis at various stages [23]. Tetracycline blocks attachment of the transfer RNA-amino acid to the ribosome, thereby inhibiting codon-anticodon interaction [28]. Erythromycin binds to the 23S rRNA molecule (in the 50S subunit) of the bacterial ribosome and blocks exit of the growing peptide chain [23,26]. Chlorampheicol binds to the 23S rRNA of the 50S bacteria ribosomal subunit and inhibits the peptidyl transferase activity and therefore elongation of the protein chain [23,25].
- Inhibition of nucleic acid synthesis: common drugs in this group include fluoroquinolones and rifamycins. Fluoroquinolones act by inhibiting DNA gyrase, an enzyme which introduces negative supercoils in the bacterial DNA prior to initiation of DNA replication [23,25,26]. Fluoroquinolones also inhibit Topoisomerase IV, which is responsible for removing the separating daughter chromosomes at the end of a round of replication [23,25,26]. Rifampin inhibit bacterial RNA polymerase, which occurs as a result of the antibiotic binding in the polymerase subunit deep within the DNA/RNA channel, causing direct blocking of the growing or elongating RNA [25,26].
- Inhibition of metabolic pathways: notable drugs in this group are sulfonamides and trimethoprim. Sulfonamides are chemical analogs of para-aminobenzoic acid and competitively inhibit dihydropteroate synthetase [23,25]. Trimethoprim inhibits dihydrofolate reductase, an enzyme that reduces dihydrofolic acid to tetrahydrofolic acid [25,26]. Both dihydropteroate synthetase and dihydrofolate reductase are important in the production of bacteria folic acid which is required for nucleotides, necessary for DNA synthesis [29].
- Interference with cell membrane integrity: polymyxin B, the notable drug in this group acts by binding to the bacterial cell membrane and altering its permeability leading to leakage of the cell [23,25,26].

Bacteria employ various mechanisms to resist the action of antibacterial drugs. Generally, these mechanisms include

- Mutational alteration of the target protein
- Enzymatic inactivation of the drug
- Preventing drug access to targets
- Permeability barriers
- Acquisition of genes for less susceptible target proteins from other species
- By passing of the target

Details of the various mechanisms of drug resistance adopted by various bacteria which is outside the scope of this review are discussed in a recent review by Davies and Davies [30]. Sometimes a bacterium may be resistant to various antibacterial agents simultaneously, a condition referred to as multidrug resistance. Many bacteria now exhibit multidrug resistance, and of more concern are superbugs such as *Stapylococcus aureus*, *Mycobacterium tuberculosis*, *Klebsiella pneumoniae* and *Clostridium difficile* [30,31]. Multidrug resistance of superbugs constitutes a major threat to public health, as it reduces treatment options, and enhances morbidity and mortality of the superbugs. Generally, multidrug resistance may occur by one of two mechanisms. Firstly, the bacteria involved may accumulate multiple resistance genes on plasmids, and each of these genes codes for resistance to a single drug [14]. Secondly, multidrug resistance may occur by increased expression of genes that encode multidrug efflux pumps, thereby extruding different types of drugs [14].

## 4. Antibiotic Discovery in the Pre-genome Era

The discovery of penicillin and its usage clinically was followed by the discovery of a large number of antibiotics from microbes in particular from members of the actinomycetes and fungi [32]. From the 1960s emergence of bacterial resistance to these antibiotics and their spread required the search for new antimicrobial agents [33]. At the time, one way that scientists approached the problem was the semi-synthetic modification of existing antibiotics that had already proven useful. Overall the semi-synthetic antibiotics were more potent and less susceptible to inactivating enzymes that cause resistance [33]. Additionally, some of these drugs expressed activity against a broad spectrum of bacteria, and toxicity problems were minimal. By the early 1980s progress in the discovery of semi-synthetic antibacterial agents had almost halted and drug resistance in bacteria was still rising. Fortunately, in the mid 1980s fluroquinolones were successfully developed by modification of nalidixic acid [34]. Not long after this, the sulphonamides were also discovered which can be traced to prontosil, a chemical compound developed by Domagk in 1935 [33]. In the past few years before the genome era, efforts aimed at discovering antibacterial agents had been fruitless, with some pharmaceutical industries investigating old antibiotic compounds that had not met potency and other requirements at the time of initial isolation.

## 5. Bacterial Genomes and Genome Sequencing

### 5.1. Bacterial Genomes

The genome of an organism refers to its entire complement of genes contained in the DNA of its chromosome (s). The bacterial genome is usually contained in a circular DNA molecule which is supercoiled and localised within the nucleoid of the cell. There are exceptions, as some bacteria have two or more chromosomes and some chromosomes may be linear. Among medically important bacteria, *Vibrio*, *Burkholderia*, *Leptospira* and *Brucella* species are those with two or more chromosomes, while *Borrelia* *burgdorferi* has its genome in a linear chromosome [35]. Most bacterial genomes are less than 5 MB, although a few, such as *Bacillus* *megaterium*, may be as large as 30 MB [36]. The major pattern in bacterial genome size is that, on average, free-living species have larger genomes than parasitic species which in turn have larger genomes than obligate pathogens. Bacterial genomes vary greatly between species in terms of nucleotide composition: The G+C (guanosine-cytosine) content may vary locally within a genome, but it is relatively uniform within a bacterial genus or species, ranging from around 25% in *Mycoplasma* spp. to around 75% in some *Micrococcus* species [35].

On the average, a typical bacterial genome has about 2,500 genes, which are maintained in a certain genomic architecture through selective pressure, rather than through a random succession of genes [35,36]. The genome of bacteria encodes all the biochemical functions that are necessary for survival. Additionally, pathogenic bacteria may carry genetic features required for virulence, while non-coding regions are also located in the bacterial genome. Characteristically, bacterial genes may be organized into operons, which refer to a group of genes located adjacent to one another, and are functionally related. An example of an operon is the lactose operon in *Escherichia coli*, which contains three genes involved in the conversion of lactose, a disaccharide into monosaacharide units- glucose and galactose [37]. 

Bacterial genomes are dynamic, and are exposed to various genetic events, including, mutations, duplications, inversions, transpositions, recombination, insertion, and deletions. Gene acquisition through horizontal gene transfer is probably the mechanism having the greatest impact on the organism’s lifestyle by conferring a novel metabolic capacity, such as acquisition of antibiotic resistance genes and virulence factors [20]. 

In some bacterial cells, apart from the genome, there may be extra chromosomal DNA molecules referred to as plasmids. Sometimes, the distinction between a megaplasmid and a second chromosome may not be clear [38]. Generally, plasmids are circular and double stranded, and replicate independently of the bacterial chromosome. Plasmids facilitate horizontal gene transfer within a microbial population of microbes and typically provide a selective advantage under a given unfavorable environmental state.

### 5.2. Principles of Genome Sequencing

DNA and protein sequencing started in the 1970s when the virus Lambda (50,000 nucleotides) was sequenced by Sanger *et al.* [39]. Around this time DNA sequencing was carried out for small genomes such as viruses and organelles, and complete sequencing of a bacterium, was not feasible because of economic and technical limitations. However, later on, sequencing of the human genome, and improvements in sequencing technologies facilitated whole genome sequencing of bacteria. The first bacterium to be sequenced was *Haemophilus influenzae* [40], and this was done by the shotgun method developed by Sanger *et al.* [39]. Briefly, the shot gun method of sequencing consists of randomly sampling and determining 500-700 nucleotide reads and then assembling them to reconstruct the sampled sequence [41]. Because the assembly process is based on finding regions that overlap, more than 1 million bases must be sequenced in order to sequence a 1-Mb genome. The mean value of the number of times each base is sequenced in a genome project is called genome coverage and is usually between 6 and 8 [41]. The method of sequencing developed by Sanger is considered the gold standard, and over the years, whole genome sequencing of many bacteria has been carried out using this method. 

Recently, next generation sequencing technologies have emerged, which are high throughput and able to generate three to four orders of magnitude more sequences and are also relatively less expensive [42]. Next generation sequencing methods employ a wide spectrum of technologies such as sequencing by synthesis [43,44], sequencing by ligation [43,44], single molecule DNA sequencing [43] and polony sequencing [45]. In recent times, the sequencing industry seems to be dominated by Illumina, who have introduced three next generation sequencing platform including GAIIx, Hiseq 2000 and Miseq [46]. These sequencing platforms employ a sequencing-by-synthesis approach [43,44]. In this method, DNA molecules and primers are attached on a slide and amplified with DNA polymerase resulting in the formation of clonal DNA colonies (DNA clusters). To evaluate the DNA sequence, four types of fluorescently labeled reversible-terminator nucleotides are added and the incorporated nucleotides are imaged. The fluorescent dye with the terminal 3' blocker, is then chemically eliminated from the DNA, allowing for the next cycle to start. 

Sequencing platforms that employ next generation sequencing technologies are being produced at a fast rate, with two major sequencing platforms introduced in 2011, namely Ion Torrent Personal Genome Machine (ITPGM) [47] and the Pacific Biosciences (PacBio) RS [48]. PacBio sequences single molecules in real time without amplification [48]. In this method, a conjugate of DNA polymerase and DNA template are attached to 50 nm-wide wells. Using nucleotide fluorescently labeled with γ-phosphate, the DNA polymerase carry out second strand DNA synthesis. Incorporation of bases during DNA synthesis is detected by means of a distinct pulse of fluorescence. ITPGM employs technological advances in semi-conductor science and non-sensitive transistors to sequence DNA [47]. This method differs from other next generation sequencing methods as polymerisation events are detected by pH changes rather than light. DNA fragments carrying specific adapter sequences are linked to a bead and then clonally amplified by emulsion PCR. The templated beads are loaded onto a chip which has proton-sensing wells that are fabricated on a silicon wafer, and sequencing is primed from a predetermined location in the adapter sequence. As bases are incorporated during the sequencing process, protons are released and a signal is detected proportional to the number of bases incorporated. Comparison of key features of the various sequencing methods described above, as well as their advantages and disadvantages are summarized in Table 1. Further advances in genome sequencing are expected in the near future as the so called third generation technologies are being developed to further increase throughput, decrease cost, and reduce the time to obtaining results. One interesting area of such sequencing methods involves microscopy based techniques such as atomic force microscopy that are used to identify the locations of nucleotides within long DNA fragments [49].

**Table 1:** Comparison of some sequencing methods.

Method	Single-Molecule Real-Time Sequencing (Pacific Bio)	Ion Semiconductor (Ion Torrent Sequencing)	Sequencing by Synthesis (Illumina)	Chain Termination (Sanger Sequencing)
Read length	2,900 bp	200 bp	50 to 250 bp	400 to 900 bp
Accuracy	99%	98%	98%	99.9%
Reads per run	35–75 thousand	up to 5 million	up to 3 billion	N/A
Time per run	30 min to 2 h	2 h	1 to 10 days,	20 min to 3 h
Cost per 1 million bases (in US$)	$2	$1	$0.05 to $0.15	$2,400
Advantages	Rapid and has longest read length.	Equipment is relatively less expensive and Fast.	Sequence yield could be very high depending upon equipment model	Long individual reads. Wide application
Disadvantages	Yield tends to be low at high accuracy. Equipment is very expensive.	Prone to homopolymer errors.	Equipment can be very expensive.	Equipment is expensive and not suitable for larger sequencing projects.
References	[46,48].	[46,47]	[43,44,46]	[39]

The objective of a genome sequencing project is a completed contiguous DNA sequence of the bacterium's chromosome (s), and the error frequency is estimated to be one error (frame shift or base substitution) in 10^3^ to 10^5^ bases. Indeed such an error rate or even a higher rate of approximately one error per gene has little effect on the usefulness of the data [50]. After a bacterial genome has been sequenced, the next thing is to annotate it. Annotation is the process by which structural, functional, and other biological information is inferred from genes or proteins, and this is based on similarity to previously characterized sequence in public databases. 

This requires bioinformatic analyses, and bioinformatics tools such as BLAST (Basic Local Alignment Search Tool) have been found very useful. The first step in the annotation process is the identification of predicted protein coding sequences, generally referred to as open reading frames. Unlike eukaryotic genomes, identification of open reading frames in bacterial and other prokaryotic genomes is remarkably accurate and also easier due to the absence of introns and also the high gene density possessed by these organisms. Only a subset of all the open reading frames in the genomic sequence actually encodes proteins, and prediction of their functions by database comparison with similar genes of known functions is the next stage in the annotation process. However, this procedure can be problematic as the functions of a large percentage of genes in some organisms are unknown [51].

Information on various organisms including strains that have been fully sequenced and annotated, and others which are still in the process of sequencing are reported at the websites of Sanger Institute [4] and National Centre for Biotechnology Information [5] Once a genome has been sequenced and annotated, basic information for understanding the biology of the organism has been obtained, and the next thing is to utilize the genome data.

### 5.3. Streptococcus Pneumoniae TIGR4 Genome: An Example of a Sequenced Genome

TIGR4 is a virulent *S. pneumoniae* strain (serotype 4, ST 205) isolated from the blood of a 30-year old male patient in Norway [52]. According to Tettlin *et al.* [52] the genome of this strain is a single circular chromosome containing 2,160,837 base pairs and 2,236 putative genes, the majority (64%) of which have been assigned a biological function. The genome has a GC content of 39.7%, and about half of the predicted proteins are most similar to proteins from other low-GC Gram-positive species. Analysis revealed that 5% of the genome is composed of repeats including insertion, BOX, and RUPS elements that may facilitate incorporation of foreign DNA into the *S. pneumoniae* chromosome and contribute to rearranging its structure. The genome encodes many ATP-dependent transporters and 30% of transporters are involved in sugar transport, which may reflect its ecological adaptation to sugar-related environments such as the oral cavity. Extracellular enzyme systems for carbohydrate metabolism provide carbon and nitrogen for the organism and facilitate colonization in host pathogen interaction. Iron and phosphate transporters as well as a 13-gene cluster involved in capsular biosynthesis may also contribute to virulence. Sixty-nine proteins are predicted to be expressed on the bacterial surface and a putative signal peptide motif identified is potentially involved in targeting these proteins to the surface of the cell. 

### 5.4. Genome Sequencing and Insights into Bacterial Pathogenesis

One of the important applications of pathogenic genome analysis is the identification of virulence genes, which can provide insights into pathogenesis of bacterial pathogens. Virulence genes are found in specific regions of the chromosomes of bacteria, forming the so called pathogenicity islands (PAIs). These regions are up to 200 kb in size, often have specific insertion sequences at their ends which facilitate their translocation and insertion between microorganisms. There are several ways of identifying PAIs or virulence genes. One approach known as the genome composition approach, involves searching for regions with DNA signatures that are distinct from other parts of the genome [51]. Related to this are clues such as tandems repeats of simple sequences found in or near certain virulence genes called contingency genes. A second approach for identification of PAIs or virulence genes is through comparative genomic analysis of closely related genomes or very different genomes of species that cause similar infections [53]. Through such comparison, new virulence factors can also be identified by finding genes that are co-regulated with known virulence factors.

Perna *et al.* [54] compared the genomes of laboratory strain *E. coli* K12 (non-pathogenic) and the pathogenic strain *E. coli* O157:H7 which causes food borne illness leading to bloody diarrhea, and sometimes kidney damage. The *E. coli* O157:H7 genome is 0.57 MB bigger than the *E. coli* K12 genome, and there are 1,387 genes present in the pathogenic strain which are absent in the non-pathogenic strain. The extra genes in the pathogenic *E. coli* O157:H7 strain are organized into pathogenicity islands (O-islands) and many of them code for toxins and other proteins that are involved in the pathogenicity of O157:H7.

The two *E. coli* genomes were later on compared with the genome of *Shigella flexneri* serotype 2a (another enteric pathogen that causes diarrhea) when it was sequenced [55]. The chromosome of the *Shigella* strain shares a common ‘backbone’ sequence ~3.9 Mb with those of *E.coli* K12 and O157. However, the *S. flexneri* chromosome carries 314 insertion elements, which is far more than that those possessed by *E. coli* O157:H7 and *E. coli* K12. Compared with the E. coli genomes, the *Shigella flexneri* serotype 2a genome has 13 translocations and inversions, which are characterized by deletion or insertion sequences and several of them, are likely to be bacteriophage-transmitted pathogenicity islands. These pathogenic features of *S. flexneri* probably explain its unique pathogenic lifestyle despite its close relationship with *E. coli* and other enterics; unlike many other enteric pathogens, *Shigella* is known to infect only humans and also has a very low infectious dose of 10–100 [1].

The genome sequences of several *S. pneumoniae* strains, including R6 (non-pathogenic and unencapsulated strain) and TIGR4 (encapsulated and pathogenic strain) were compared to evaluate virulence genes associated with this organisms which causes meningitis, pneumonia, and septicaemia [56]. The striking difference observed between the two strains is the high density of capsular genes in TIGR4 and the complete absence in R6, which confirms the capsule of *S. pneumoniae* as its major virulence determinant. Several other virulence genes including neuraminidase A, choline binding protein A, and immunoglobulin A1 protease were also present in TIGR4 but not R6. This indicates that other factors are required besides the capsule for full virulence.

*Streptococcus pyogenes* is part of the normal bacterial flora and causes benign pharyngitis and also invasive disease such as scarlet fever. Genome sequencing of *S. pyogenes* has identified several virulence genes such as C5a peptidase, streptolysin O, and streptolysin S [1,57]. Comparison of the genomes of 86 serotype M3 *S. pyogenes* pharyngitis strains with those of 215 invasive M3 strains from Ontario, Canada showed that the two groups of strains were genetically similar [58,59]. This shows that the ability of *S. pyogenes* to cause invasive disease is not restricted to specific strains, an observation which has also been reported for other bacterial pathogens such as *S. pneumoniae* [60]. 

Whole genome sequencing approaches have been very important in elucidating transmission of bacterial pathogens. In a genomics study of *Burkholderia dolosa* among chronic cystic fibrosis patients, Lieberman *et al.* [61] used the chronology of mutation patterns to differentiate donors from recipients in the transmission network, and to infer multiple transmissions from the air to the bloodstream within patients. Similarly, Reeves *et al.* [62] were able to distinguish zoonotic transmission from human to human transmission in a persistent *Escherichia coli* infection of members of a household. Based on whole genome sequencing and analysis of single nucleotide polymorphism differences of non-typhoidal salmonella strains, Okoro *et al.* [63] distinguished multiple transmission events from relapsing infections in fourteen Malawian patients. Relapsing accounted for 78% of recurring infections, and the occurrence of relapsing and multiple infection in the same patient was rare [63].

The above selected examples span different categories of bacterial pathogens and highlight some of the contributions of genome sequencing to our understanding of bacterial pathogenesis. Further insights into bacterial pathogenesis are expected as more and more strains of a given pathogen, from different disease and ecological states are being sequenced. With the advances in genome sequencing and its decreasing cost, it is likely that genome sequencing would be used routinely in diagnostic bacteriology and also surveillance of bacterial pathogens.

### 5.5. Genome Sequencing and Insights into Development of Antibiotics

Another important application of genome sequence data is the discovery of antibiotic targets for development of novel antibiotics. This important application cannot be overemphasized, considering the current trend of increasing antibiotic resistance, especially multiple drug resistance of superbugs [30]. Next generation sequencing platforms such as PacBio can provide methylation data, which could be useful in designing antibiotics and understanding antibiotic resistance [48,64]. For example, sequencing of *Stapylococcus aureus* isolates collected from across the globe provided unprecedented insights into antibiotic resistance of this superbug, including resistance mechanisms, microevolution and molecular epidemiology [65]. Resistance is more likely to happen when newly designed antibiotics are chemically similar to previous ones already rendered ineffective. Therefore ideally, new antibiotics should have novel mechanisms of action, which is the ultimate goal of the genome sequencing approach to discover novel antimicrobials. Current antimicrobial agents target a small fraction of the bacterial genome indicating good prospects for discovery of novel antibacterial drugs [66]. Glass *et al.* [66] summarized the essential attributes of good drug targets in the genomes of bacteria: Firstly, good drug targets must be essential for viability or required for disease; secondly, they must be unique to bacteria or at least significantly different from orthologous genes in humans; and thirdly, for broad spectrum antibiotics, the targets must be present in key pathogenic bacteria. Identification of such targets is mainly done by comparative genomic analysis using bioinformatics approaches such as sequence homology, structural homology, cluster analysis and motif analysis [67,68,69]. Once potential drug targets are identified, they must be evaluated experimentally, using gene essentiality testing methods, followed by testing large chemical libraries of potential antimicrobials, and modifying candidate molecules to improve their efficacy and reduce toxicity [70]. 

Following publication of the *S. pneumoniae* genome, the organism has been used as a genomics platform for discovery of novel antibiotic targets. This is due to its enormous medical importance [71,72], as well as, its utility for traditional microbiology and genomics based experimentations, based on its natural capacity to be transformed by exogenous DNA [73]. In one study, 113 conserved essential genes were identified in *S. pneumoniae* by disrupting over 300 candidates using a suicide vector [74]. In another study, 36 essential genes for growth were identified among 144 open reading frames with previously uncharacterized functions [75]. In a comprehensive study, Song *et al.* compared the genome of *S. pneumoniae* R6 with those of *Bacillus subtilis*, *Enterococcus faecalis*, *E. coli*, and *Staphylococcus aureus*, and selected 693 candidate target genes [76]. The genes were selected on the basis of >40% amino acid sequence identity to the corresponding genes in at least two of the other species [76]. The 693 genes were disrupted and 133 were identified to be essential for growth. Overall, more than 200 essential genes of *S. pneumoniae* have been identified, and many of these genes have been catalogued by Song *et al.* [76]. Freiberg *et al.* studied 27 originally uncharacterized genes similar to proteins derived from genomes of phylogenetically diverse pathogenic bacteria such as *H. influenzae*, and Gram positive organisms including, *Streptococcus* spp., *Staphylococcus* spp., and *Enterococcus* spp. [77]. To evaluate whether these 27 genes were essential genes, they were deleted in *E. coli*, and 6 of them (YgbP, YgbB, YchB, KdtB, YjeE, and YqgF) were found to be essential for growth. Interestingly, some of these genes had also been found to be essential in *Mycoplasma genitalium* and *Bacillus subtilis* [78]. Since the essential genes reported by Song *et al.* [76] and Freiberg *et al.* [77] were identified in a variety of organisms including, Gram positive and Gram negative bacteria, they represent suitable targets for discovery of broad spectrum antibiotic. Several other investigators have also identified antibiotic targets through genome sequence data of bacteria [79,80].

It is important to mention that though antibiotics targets have been discovered through genome sequencing efforts, no antibiotics have reached the marketplace via this route. Progress towards development of lead compounds that inhibit the antibiotics targets have been very slow. Using the example of the pharmaceutical company GlaxoSmithKline, 67 high throughput sequencing antibacterial targets were investigated between 1995 and 2001, and only 5 resulted in lead compounds [79]. Part of the problem may be attributed to the fact that chemical libraries have been biased towards meeting Lipinski’s ‘rule of five’, a chemical algorithm used to predict drug-likeness [81]. The problem with Lipinski’s ‘rule of five’ is that many existing antibiotics do not conform to it and therefore its applicability to antibiotics is not conclusive [79]. Another major issue is that, though several conserved antibiotic targets had been discovered through genome sequencing, antibiotics that bind to the targets may not be able to penetrate the bacteria or may be removed by efflux [79,82]. There is also the problem of high susceptibility of single targets to mutational resistance. A good example is peptide deformylase, a genomics a derived target [79,83]. Though suitable lead compounds that inhibit this enzyme have been developed, they have a high tendency to generate mutants and therefore antibiotic development related to this target has not gone progressed beyond clinical trials [84,85].

The future of antibiotic discovery looks a bit uncertain. Many pharmaceutical companies are less interested in antibiotic development partly because of cost and the fact that multiple antibiotic resistance is developing at a fast rate. With the disappointment of genomics in antibiotic discovery, many pharmaceutical companies are shifting from a genomics approach to other strategies previously used in the industry such as natural product screening. Recently, Zhang *et al.* [86] used genome sequence data of *Streptomyces* sp. W007 and natural product screening to identify novel angucyclinone antibiotics. This provides good evidence for the interaction between genomic analysis and traditional natural product isolation research. Thus, it is possible that in the long-term, genome sequence information will be useful to antibiotic discovery, but probably not in the way originally thought (ie as a short cut to target selection and subsequently development of novel antibiotics).

## 6. Conclusions

As shown by this review report, the advent of genomics has greatly advanced our understanding about the mechanisms by which bacteria cause disease. This coupled with our understanding of other biological information such as ecology of the pathogens (also highlighted by genomic data) sets the stage for design of effective therapeutic interventions against bacterial diseases. Bacterial genome sequencing has also helped to identify new drug targets, which can be used in the design of novel antibiotics. However, so far, antibiotics have hardly reached the marketplace via the genome sequencing route.
